# Enhancing professional nurses’ preparedness and collaborative practices in managing gender-based violence: Insights from selected South African university campus health clinics

**DOI:** 10.4102/hsag.v31i0.3103

**Published:** 2026-02-19

**Authors:** Siphesihle D. Hlophe, Vasanthrie Naidoo, Nellie Naranjee

**Affiliations:** 1 Department of Nursing, Faculty of Health Sciences, Durban University of Technology, Durban, South Africa

**Keywords:** gender-based violence, higher education institutions, nursing education, professional nurses, curriculum development, victim support

## Abstract

**Background:**

Gender-based violence (GBV) is a critical public health issue, particularly in South African Higher Education Institutions (HEIs). This study explores professional nurses’ preparedness to manage GBV cases and identifies gaps in their formal training.

**Aim:**

To examine professional nurses’ experiences and perceptions of their competence, interdisciplinary collaboration, and use of reporting, referral, and institutional resources in managing GBV at campus health clinics in HEIs.

**Setting:**

Research was conducted at two universities, Institution One and Institution Two, focusing on professional nurses employed at campus health clinics.

**Methods:**

An exploratory-descriptive qualitative design was used, with data gathered through semi-structured interviews with five professional nurses.

**Results:**

Four themes emerged: (1) enhancing interdisciplinary collaboration through reporting and referrals; (2) challenges in managing GBV victims; (3) professional training and knowledge on GBV; and (4) nurses’ competence in handling GBV cases. This article emphasises themes one and four. Findings reveal the need for comprehensive GBV modules in nursing education and ongoing in-service training. Nurses reported challenges including limited victim information, systemic barriers and complex documentation.

**Conclusion:**

Integrating GBV education into nursing curricula alongside continuous professional development and institutional support is essential to equip healthcare professionals to provide empathetic, comprehensive care to victims.

**Contribution:**

This study offers insights for curriculum reform and policy development aimed at reducing GBV and promoting awareness and advocacy within HEIs.

## Introduction

Gender-based violence (GBV): It is not just a headline; it is a shadow that touches every corner of the globe, regardless of culture or income. The United Nations calls it a fundamental violation of human rights, a public health crisis that demands our urgent attention (United Nations 2020). And within the hallowed halls of healthcare and higher education, GBV presents a unique challenge, weaving its way into policy, patient care and the very fabric of learning (United Nations Women 2021). Professional nurses stand on the front lines of this battle, particularly within higher education institutions (HEIs). They are not merely caregivers; they hold a strategic role in developing the next generation of healthcare professionals and in shaping policies that safeguard survivors of GBV. Enhancing nurses’ preparedness has the potential to improve outcomes for survivors of GBV and contribute to broader societal change (Garcia-Moreno et al. 2014:8). Although comprehensive statistics on GBV in South African HEIs are limited, existing data indicate a troubling prevalence. Approximately 10% of all reported GBV cases in South Africa occur within HEIs. Research highlights that GBV, including sexual violence, is a significant problem affecting both students and staff (Brink et al. 2021:47). While many acts of GBV, particularly rape, are reportedly committed against university students, there is currently no nationally representative data detailing the full extent of GBV on South African HEI campuses (Makhene 2022:235).

In South Africa, nurses are often the first lifeline for GBV survivors, shouldering the responsibility of recognising the signs, providing immediate care and connecting individuals with vital support services (Oparinde & Matsha 2021:73). Effective GBV management demands a holistic approach, understanding the physical, emotional and psychological scars that survivors carry (Klazinga, Artz & Müller 2020:15). Yet, despite the critical need, a global gap persists: nursing curricula often fall short in providing adequate training on domestic abuse, hindering nurses’ ability to effectively manage these sensitive cases (Alshammari, McGarry & Higginbottom 2018:44). The World Health Organization (WHO) has stressed the importance of focused clinical training for all healthcare practitioners (WHO 2019).

Closing this educational gap requires weaving GBV education into the core of nursing programmes. Equipping nurses with skills in forensic evidence collection and survivor education is paramount (Sepeng, Makhado & Sehularo 2022:127). But in South Africa, the Bachelor of Nursing undergraduate degree programme, while comprehensive, allocates limited credits to the Social Science module – a mere 14 out of 480 – crucial for understanding the social forces that influence health (South African Nursing Council [SANC] 2020:1). This theoretical focus often lacks the practical application needed to assess essential skills and competencies honestly. Even with theoretical knowledge, professional nurses face numerous hurdles: insufficient training, limited resources, pervasive stigma and systemic roadblocks (Mathews & Abrahams 2020:2). Integrating work-integrated learning and co-curricular activities focused on GBV management is essential for creating well-rounded nursing professionals.

This study steps into this critical space, investigating the preparedness of professional nurses in selected HEIs to recognise and respond to GBV. It is about uncovering the gaps in knowledge, skills and resources and exploring recommendations for enhancing nursing curricula. By identifying these gaps, as perceived by professional nurses, this research contributes to the ongoing efforts to mitigate the impact of GBV in educational and healthcare settings. Therefore, this study aimed to explore professional nurses’ experiences and perceptions regarding their competence, interdisciplinary collaboration and use of reporting, referral and institutional resources in managing GBV within campus health clinics at HEIs.

## Research methods and design

### Study design

The research design for this study was exploratory-descriptive qualitative. A qualitative method aims to give precedence to the research participants’ viewpoints and highlight the subjective meaning, behaviours and circumstances of those being investigated. The selection of this design allowed the researcher to achieve an understanding of the experiences and perspectives of participants’ knowledge and training related to recognising and responding to GBV.

### Study setting

South Africa has some of the highest rates of GBV in the world, and statistics often reveal the scale of the problem in terms of incidents per month. According to the South African Police Service Crime Statistics (2023), there are approximately 3000 women murdered because of domestic violence and femicide, averaging 250 per month (South African Police Service 2022:15). The research was conducted at two universities, Institution One and Institution Two, and focused on the experiences and perceptions of professional nurses’ knowledge and training related to recognising and responding to GBV employed at the Campus Health Clinics. These clinics are primarily established to provide primary healthcare services to students and staff at their institutions. They focus on promoting healthy lifestyles, preventing illness and offering opportunities for student learning and community engagement. Additionally, these clinics play a crucial role in addressing GBV on campus. Strategically located within university grounds, they offer targeted awareness campaigns, support services and management resources to reduce victimisation and improve survivors’ outcomes. In this way, campus health clinics serve as general health centres and vital hubs in the fight against GBV.

### Study population and sampling strategy

The target population for this study comprised professional nurses employed at the Campus Health Clinics of Institution One and Institution Two. The study utilised purposive sampling to select participants directly involved in managing, combating or creating awareness of GBV. All eligible professional nurses at Institution One and Institution Two were invited to participate, ensuring a maximum variation sample and maximising the potential to gather rich, insightful data relevant to the study’s objectives. The proposed study population included five to eight professional nurses; however, only five out of eight participants were willing to participate in the study.

### Recruitment

Professional nurses at Campus Health Clinics within Institution One (IG – 4773) and Institution Two (UWCRP681567) were recruited through a collaborative process initiated by contacting the Heads of Departments (HoDs) for nursing. After establishing rapport with the HoDs, the researcher, guided by Gray and Grove’s (2021:374) recruitment strategies, outlined the study’s objectives and inclusion and/or exclusion criteria. Professional nurses included in the study met specific inclusion criteria: they were employed at the Campus Health Clinics of Institution One and Institution Two in the City of Cape Town. They were registered with the SANC. These nurses had been directly involved in managing or responding to GBV cases or had participated in GBV-related campaigns or activities aimed at raising awareness within the campus community. Additionally, they had at least 6 months of employment at the time of data collection and were willing to participate in interviews. The exclusion criteria ruled out professional nurses who were not employed at the Campus Health Clinics of the specified institutions, enrolled nurses or nursing assistants who did not meet the required qualification level, those employed for less than 6 months, nurses not involved in GBV-related activities or campaigns and those who declined participation.

### Data collection

Data were collected through semi-structured interviews designed to explore participants’ perspectives in depth. The interview guide, informed by Charmaz’s (2014:65) insight that open-ended questions foster rich discussions, consisted of two parts. The first part introduced the study by outlining its topic and objectives, while the second part featured open-ended questions encouraging detailed responses and follow-up. These questions were carefully aligned with the study’s aims, focusing on professional nurses’ experiences and perceptions of their knowledge and training in recognising and responding to GBV.

Interviews were then scheduled based on each nurse’s availability and preference for either face to face or online formats. Ultimately, all interviews were conducted online via Microsoft Teams, respecting participants’ wishes. Each interview lasted between 30 min and 45 min and was arranged to minimise disruption to the nurses’ daily duties.

The interview guide, pre-tested for clarity and relevance, was used consistently to explore nurses’ experiences and perceptions regarding their knowledge and training on GBV within higher education settings. With participants’ consent, all interviews were audio-recorded to capture responses and verbal cues accurately. These recordings were later transcribed with permission.

### Data analysis

Data analysis followed Tesch’s method (1992:141) to understand the conceptual and contextual issues emerging from the transcribed interviews. The process began with thoroughly reading all transcripts to gain a general impression of the collected data, followed by annotating thoughts and initial ideas in the margins. A comprehensive list of topics was then generated, with similar issues clustered together and abbreviated into codes corresponding to specific data segments. Descriptive wording was used to transform these topics into overarching themes, which were then grouped into related categories. Preliminary data analysis involved assembling data from each category to identify emerging themes, re-describing meaning units in psychological language to connect them to the study’s subject and synthesising primary themes and patterns about the research questions. Throughout the analysis, emphasis was placed on maintaining the integrity of the participants’ perspectives while streamlining irrelevant information. All data were securely managed using password-protected OneDrive and locked physical storage, with a plan for secure destruction 5 years after the study’s completion.

### Ethical considerations

Participants were provided with a detailed information letter explaining the study’s aim, objectives and significance. Informed consent was carefully obtained, highlighting the confidentiality and anonymity assured through participant coding. They were informed of their right to withdraw at any point without consequence and reassured that their participation was entirely voluntary. Prior to conducting interviews, ethical clearance was secured from each university’s ethics office, and coordination with the HoDs at the Campus Health Clinics ensured participant availability.

This research strictly followed ethical guidelines to protect participants’ rights and well-being. Ethical approval was granted by the institution’s Research Ethics Committee (IREC 073/24), alongside institutional permissions from Institution One (IG – 4773) and Institution Two (UWCRP681567). Approval was also obtained from the Campus Health Clinics. Informed consent was confirmed before participants participated in semi-structured interviews, reflecting the core principles of the Belmont Report: respect for persons, beneficence and justice.

Throughout the study, considerable care was taken to comply with relevant regulations and to shield participants from any physical or psychological harm. They were informed that they could pause or discontinue interviews at any time without penalty. Interviews were conducted in private, secure online environments to maintain confidentiality and protect participants’ privacy. All data collected were anonymised and securely stored, accessible only to the researcher and supervisors.

Key ethical considerations included obtaining informed consent, maintaining confidentiality and anonymity and upholding beneficence and non-maleficence. Participants received full disclosure about the study’s nature, aims, data collection methods and their right to withdraw at any time. Confidentiality was preserved by restricting access to electronic data stored on password-protected OneDrive and securely managing physical documents. The principles of beneficence and non-maleficence were upheld by ensuring participants faced no harm – such as distress from sensitive questions or breaches of confidentiality – and by prioritising their well-being throughout the research process. Additionally, the study embraced the principle of justice by ensuring fair and equal treatment of all participants, with equal consideration given to their contributions.

To ensure the trustworthiness of this qualitative research, several strategies were implemented following Lincoln and Guba’s (1985) framework, focusing on credibility, transferability, dependability and confirmability. Credibility was enhanced by recording all interviews, using direct quotes to represent participants’ voices and maintaining prolonged engagement in the research setting. Transferability was addressed through thick descriptions of the research context, methodology and findings, enabling readers to assess the applicability of the study’s insights to other settings. Dependability was achieved through data triangulation, comparing interview data with curriculum documents and involving research supervisors as independent coders to verify data and analysis. Confirmability was supported by detailing the data examination process, ensuring that interpretations represented the participants’ voices rather than researcher bias and preserving audio recordings and field notes for future auditing.

## Results

Among the professional nurses interviewed, a majority, 80%, were female, while the remaining 20% were male. Age demographics were varied, with 40% of the participants aged between 31 and 40 years, and equal proportions of 20% in the 20–30, 41–50 and over 51 age brackets (Table 1). Educational qualifications revealed that 80% of the nurses had achieved postgraduate degrees, while 20% held undergraduate degrees (Table 2). This diverse range of ages and educational backgrounds reflects a broad spectrum of experiences and perspectives within the professional nursing interviews.

**TABLE 1:** Professional nurses’ demographics.

Demographic	No of participants
**Gender**	
Male	1
Female	4
Prefer not to say	0
**Age (years)**	
20–30	1
31–40	2
41–50	1
51 >	1
Prefer not to say	0
**Level of study/qualification**	
Undergraduate	1
Post-graduate	4
Masters	0
Doctorate/PhD	0
Prefer not to say	0

**TABLE 2:** Professional nurses’ individualised demographics.

Participant code	Institution code	Gender	Age (years)	Level of study
Participant 1	Institution 1	Female	58	Postgraduate Diploma
Participant 2	Institution 1	Female	39	Postgraduate Diploma
Participant 3	Institution 1	Female	45	Postgraduate Diploma
Participant 4	Institution 2	Male	32	Undergraduate Diploma
Participant 5	Institution 2	Female	28	Postgraduate Diploma

Four primary themes emerged from the semi-structured interviews with five professional nurses in the main study: (1) enhancing interdisciplinary collaboration and support through effective reporting, referral and resource utilisation; (2) challenges encountered in managing GBV victims; (3) professional training and knowledge development in managing GBV and (4) competence and skill of professional nurses when dealing with GBV matters (Table 3). This article focuses specifically on the first and fourth themes because they directly relate to practical strategies for improving patient care and nurse preparedness within campus health clinics. These themes offer valuable insights into how collaborative approaches and individual competence can be strengthened to better support victims of GBV. The first theme includes seven subcategories, while the fourth comprises five subcategories, all richly supported by participants’ narratives and explored in detail in this section.

**TABLE 3:** Major themes and sub-themes.

Themes	Sub-themes
1. Enhancing interdisciplinary collaboration and support through effective reporting, referral and resource utilisation	1.1 Consent and obligation to report issues of GBV 1.2 Referral of GBV victims to specialised centres for treatment and management 1.3 Institutional support and policies for GBV victims 1.4 Collaboration with other professionals within the multidisciplinary team 1.5 Role of employee wellness programmes in the management of GBV 1.6 Guidelines and protocols related to GBV 1.7 Referral systems related to GBV
2. Competence and skill of professional nurses when dealing with GBV matters	2.1 On-the-job learning related to GBV 2.2 Professional nurse experience 2.3 Using empathic and non-judgemental approaches in managing GBV victims 2.4 Utilising effective communication and listening skills in managing GBV victims 2.5 Understanding human rights and ethical policies related to GBV victim management

*Source:* Hlophe, D.S., 2025, Thesis submitted in fulfilment of the requirements for the Doctor of Nursing at the Durban University of Technology, Durban, South Africa.

GBV, gender-based violence.

### Theme 1: Enhancing interdisciplinary collaboration and support through effective reporting, referral and resource utilisation

This theme focuses on professional nurses’ practices and challenges in reporting and managing GBV cases, highlighting the importance of victim consent, mandatory reporting in certain situations and collaboration with specialised support services. Seven sub-themes emerged from this area, namely:

#### Sub-theme 1.1: Consent and obligation to report issues of gender-based violence

Nurses emphasise the importance of obtaining permission from victims before reporting cases to the police and social workers. However, they also acknowledge their duty to report specific cases, especially involving children, regardless of consent. The following excerpt from the participant interview attests to this:

‘I do interviews to get their consent and ask them if I can phone the police. I can help you with the social workers, and I will inform the person that I must report this case, even if you don’t want me to. But anyway, if it’s a child, then I will say I must report that, whether you don’t want me to report it, it is my duty.’ (Professional Nurse, Participant 1, Female, 58 years)

‘I’m not sure if we have policies on that. The resources are central. We are connected to them. We have to report all GBV cases. We as professionals must report it even if it happened a long time ago.’ (Professional Nurse, Participant 3, Female, 45 years)

This sub-theme highlights a clear tension between respecting victim autonomy and fulfilling mandatory reporting duties, particularly in cases involving children. While nurses strive to obtain consent before involving authorities, the obligation to report certain cases regardless of consent reveals a gap in balancing ethical considerations with legal responsibilities. This underscores a need for clearer policies and better support to help nurses navigate these complex situations confidently and consistently.

#### Sub-theme 1.2: Referral to specialised centres for treatment and management

Thuthuzela Care Centres (TCCs) are frequently mentioned as specialised facilities for handling sexual abuse cases, providing shelter and offering comprehensive support services. Thuthuzela Care Centres are one-stop facilities located at public hospitals or clinics, primarily in communities with high rape incidence, and are part of South Africa’s anti-rape strategy. They are government facilities, specifically led by the National Prosecuting Authority’s Sexual Offences and Community Affairs Unit. The following participant quotes are noted as follows:

‘Thuthuzela Healthcare Centres near institutional 1. I have numbers here; it’s where they deal with gender-based violence and people of all ages. They also provide shelter, say, now it was a court case, and that person needs to be protected. This shelter only provides for 72 hours; the person must be placed out of the shelter.’ (Professional Nurse, Participant 1, Female, 58 years)

‘… social workers are usually at the facilities where we refer to, maybe at the hospital. The social workers are also at the Thuthuzela Centres; they need to be referred to if it’s a case like that. Thuthuzela is not only just for rape crisis, but they also have other services for gender-based violence. They have two different hubs there.’ (Professional Nurse, Participant 1, Female, 58 years)

This sub-theme reveals the critical role of specialised centres like TCCs in providing immediate shelter and comprehensive support for GBV victims. However, the limited duration of shelter and the reliance on referrals highlight gaps in continuity of care and resource availability. Strengthening the capacity and accessibility of these centres, along with clear referral pathways, is essential to ensure sustained support for survivors beyond initial crisis intervention.

#### Sub-theme 1.3: Institutional support and policies for gender-based violence victims

Institutional policies and support systems like the Centre for Student Counselling and Development (CSCD) are crucial for counselling and further referrals. Participants have noted this in the following voices:

‘If it is here on campus, we will refer to Employee Wellness. They can provide services such as social workers … referrals on campus, Centre for Students Centre and Development (CSCD), our counsellor and psychologist.’ (Professional Nurse, Participant 2, Female, 39 years)

‘We assess clients and, upon talking and realising that maybe there isn’t enough information, or maybe there’s a lot of trauma, or even if there’s not a lot of trauma, we still refer them for proper counselling to individuals who have been properly trained for counselling. So often we refer them to social workers. Also to psychologists, so that maybe they can have sessional counselling with them.’ (Professional Nurse, Participant 4, Male, 32 years)

‘For us it’s mainly just managing what the patient comes presenting with and If not if there’s usually a need to escalate to other multidisciplinary, like to refer to a doctor for further assessments and evaluation to or to refer to the police services if the victim wants to open a case because that’s all up to them?.’ (Professional Nurse, Participant 5, Female, 28 years)

This sub-theme underscores the importance of institutional support systems and policies in facilitating counselling and multidisciplinary referrals for GBV victims. While these structures exist, the variability in how referrals are made and managed points to gaps in standardised protocols and consistent implementation. Enhancing clear guidelines and training for staff could improve coordination and ensure victims receive timely and appropriate support across services.

#### Sub-theme 1.4: Collaboration with other professionals within the multidisciplinary team

There is a strong emphasis on collaborating with social workers, psychologists and police services to provide holistic care to GBV victims. The following statements reflect this perspective:

‘They fear being labelled as sexually violated individuals because there’s also an element of not trusting. So if you refer them to a psychologist, maybe to a psychologist. And then maybe they know that psychologist, or maybe they know the social worker personally. And then maybe they know that psychologist, or maybe they know the social worker personally. So it’s an issue of whether I go there. She will know, or he will know, the person who will know that I’ve been sexually violated. And then it also gives the same stigma decision that I mentioned, that they fear they have a fear of being labelled as the victim.’ (Professional Nurse, Participant 4, Male, 32 years)

‘No, not yet. We don’t have any of these specific people that we work with … but if we have victims of sexual abuse, we do refer them to Karl Bremer hospital at the Thuthuzela Care Centre. Like I said, for physical abuse, I would advise you to open a case at the police station. I can advise them to contact the employee wellness people if they don’t want to. So, there are a lot of different possibilities for you.’ (Professional Nurse, Participant 2, Female, 39 years)

This sub-theme highlights the vital role of multidisciplinary collaboration in GBV care but also exposes challenges around trust, confidentiality and stigma that hinder effective referrals. The lack of established networks between nurses and other professionals indicates a gap in integrated team structures and communication channels. Building stronger, confidential partnerships within multidisciplinary teams could reduce barriers for victims and improve the quality of holistic support.

#### Sub-theme 1.5: Role of employee wellness programmes in the management of gender-based violence

These programmes are highlighted as resources for addressing work-related and personal issues, including GBV, by providing access to trained staff and social workers. The following quotes clarify how participants felt:

‘If it is here on campus, we will refer to Employee Wellness. They can provide services such as social workers … on campus, it would be CSCD, our council and a psychologist.’ (Professional Nurse, Participant 2, Female, 39 years)

This sub-theme points to employee wellness programmes as valuable resources for supporting GBV victims, particularly within campus settings. However, reliance on these programmes may be limited by awareness, accessibility or capacity constraints. To strengthen GBV preparedness, expanding the reach and visibility of employee wellness services and ensuring they are equipped to handle GBV cases effectively is essential.

#### Sub-theme 1.6: Guidelines and protocols related to gender-based violence

Participants utilise the PACK guidelines to manage GBV cases. These guidelines provide a structured approach to care although they are not universally available or used. Participants stated the following:

‘I have the PACK guidelines. I’m not just a professional nurse; I’m a clinical nurse practitioner, and we get trained according to the guidelines we follow. So, we follow the government’s guidelines. I was trained in those guidelines, and that’s how I know what to do, and I use the PACK daily. It’s a Western Cape guidelines practical approach to care kit.’ (Professional Nurse, Participant 3, Female, 45 years)

‘We don’t have any policies in place for now, we just have guidelines on how to manage the gender based violence victims, which is to assess and then you refer.’ (Professional Nurse, Participant 5, Female, 28 years)

This sub-theme reveals that while PACK guidelines offer a valuable framework for managing GBV cases, inconsistent availability and use limit their effectiveness. The absence of formal policies and reliance on guidelines indicates a gap in standardised, enforceable protocols. Strengthening policy development and ensuring consistent training and access to guidelines would improve nurses’ preparedness and the quality of GBV care.

#### Sub-theme 1.7: Referral systems related to gender-based violence

Many participants emphasise the importance of referral systems to specialised centres and professionals, such as psychologists and social workers, to provide comprehensive care to GBV victims. The following excerpt from participants’ interviews attests to this:

‘As I mentioned now, for our referrals of GBV cases, we have phone numbers of the different Thuthuzela centres in Cape Town, the Lifeline, ChildLine and child emergency helpline, emergency number 10111 number, a helpline for stop gender-based violence, the GBV centre here near the institution 1, I have Red Crisis number.’ (Professional Nurse, Participant 1, Female, 58 years)

‘If you don’t feel comfortable with that, refer to somebody in the facility or phone your colleague. As I said, we have the ChildLine, the gender-based violence, Thuthuzela Centres, and the Red Crisis Shelter. So, we have all the resources and do not need to be ignorant.’ (Professional Nurse, Participant 1, Female, 58 years)

‘I have no counselling skills regarding that. We are not trained in counselling as a professional nurse; we refer to our colleagues to manage the patient further … the psychologists or the centre. There is a dedicated centre in our area where we refer cases of gender-based violence, and they’ll manage them. The psychologists are part of the organisation.’ (Professional Nurse, Participant 3, Female, 45 years)

‘I wouldn’t know if we do refer them or if I should come up if I should come across someone. I would, you know, just be able to advise them where to go for counselling. For physical abuse, they can make a report by going to the police station to open a case. For mental and emotional abuse, they can go and see a psychologist or social worker.’ (Professional Nurse, Participant 2, Female, 39 years)

This sub-theme underscores the critical role of referral systems in connecting GBV victims to specialised support services. While nurses are generally aware of available resources and helplines, inconsistencies in counselling skills and uncertainty about referral processes reveal gaps in training and system coordination. Enhancing referral protocols and ensuring nurses are confident in guiding victims through these pathways would strengthen the overall response to GBV.

### Theme 2: Competence and skill of professional nurses when dealing with gender-based violence matters

This theme explores professional nurses’ experiential learning and approaches to managing GBV cases, emphasising empathy, effective communication and integrating human rights and ethical considerations. Five sub-themes emerged from this focus, namely:

#### Sub-theme 2.1: On-the-job learning related to gender-based violence

Participants often rely on practical experience handling cases in their professional settings. This includes learning from colleagues and through direct interaction with victims. The following extract from participants’ interviews confirms this:

‘I would say that experience has helped a lot. Unlike somebody who has just come and they don’t know, they don’t have the know-how of working with gender-based violence victims, and I would say again that.’ (Professional Nurse, Participant 4, Male, 32 years)

‘It’s just skills that come automatically when the person sits in front of you; you have to communicate, you have to interview, you have to be a good listener, you can’t give advice, you must let the patient decide, we can’t decide for the patient. But when it comes to gender-based violence, we have a responsibility to report.’ (Professional Nurse, Participant 1, Female, 58 years)

This sub-theme highlights that while practical, on-the-job learning is central to nurses’ preparedness in handling GBV cases, it also reveals a gap: much of the knowledge and skills are acquired informally and through experience rather than structured training. This reliance on experiential learning may result in inconsistencies in care and preparedness, underscoring the need for more formalised education and protocols to better equip nurses for these complex situations.

#### Sub-theme 2.2: Professional nurse experience

Some participants have had exposure to specialised units, such as Rape Crisis Centres, which have provided them with practical insights into managing GBV cases. Participants confirmed this with the following statements:

‘… I think I worked at the in my undergrad a couple of hours at the Rape Crisis Centre. So, I was exposed to it undergrad a little, [*uh*] I don’t know, it’s something that needs to be investigated, updated maybe to be part of the psychiatric placements.’ (Professional Nurse, Participant 3, Female, 45 years)

This sub-theme underscores the value of specialised exposure, like placements in Rape Crisis Centres, in enhancing nurses’ understanding of GBV management. However, it also points to a gap in formal training opportunities, as such experiences are often limited or optional. This suggests integrating comprehensive GBV-focused training into standard nursing curricula to ensure broader and more consistent preparedness.

#### Sub-theme 2.3: Using empathic and non-judgemental approaches in managing gender-based violence victims

Participants highlighted the importance of approaching GBV victims empathetically and without judgement. This approach is crucial in building trust and effectively managing cases. The following excerpt from participants’ interviews supports this:

‘As when you approach someone who has been, let’s say, maybe sexually violated, I’ll put it that way. You do not judge them; you do not impose the idea of it being their fault that such happened.’ (Professional Nurse, Participant 4, Male, 32 years)

‘The content would be that we are taught in different ways of approaching GBV victims. We are also trained on how to conduct ourselves in front of them. We shouldn’t joke around with sensitive issues. And also, we must be welcoming; you must show support so they can trust us. They must also be. We must also be transparent when conducting this research with them and make them understand that it can happen to anyone anytime, since our country is unsafe.’ (Professional Nurse, Participant 4, Male, 32 years)

‘And we also review it as we approach it in a non-judgmental manner in which, as professional nurses, we were trained to be empathetic to individuals subjected to GBV.’ (Professional Nurse, Participant 5, Female, 28 years)

This sub-theme highlights the critical role of empathy and non-judgemental attitudes in building trust and supporting GBV victims effectively. Despite training on these approaches, the deeper gap lies in consistently applying them in practice, especially given the sensitive and complex nature of GBV cases. Strengthening ongoing support and reflective practice could help nurses maintain these essential qualities under challenging circumstances.

#### Sub-theme 2.4: Utilising effective communication and listening skills in managing gender-based violence victims

Effective communication and listening skills are essential for understanding the needs of GBV victims and providing appropriate support. Participants stated the following:

‘It’s just skills that come automatically when the person sits in front of you; you have to communicate, you have to interview, you have to be a good listener, you can’t give advice, you must let the patient decide, we can’t decide for the patient. But when it comes to gender-based violence, we have a responsibility to report.’ (Professional Nurse, Participant 1, Female, 58 years)

‘The only way I can manage it is if patients trust me to tell me about it. So, I must be open so that if they disclose to me, I need to know the next steps, and they need to trust me to refer them to the right path. As a professional nurse, I can follow the PACK guidelines; these guidelines have a section that will guide you as a professional nurse on what to do with the abused or traumatised client if they were sexually abused.’ (Professional Nurse, Participant 3, Female, 45 years)

This sub-theme emphasises that effective communication and active listening are fundamental to gaining victims’ trust and delivering appropriate care. However, the gap lies in ensuring nurses possess these skills and have clear, accessible protocols like the PACK guidelines to guide their responses. Strengthening skill development and standardised procedural knowledge is essential for consistent and confident GBV case management.

#### Sub-theme 2.5: Understanding human rights and ethical policies related to gender-based violence victim management

Incorporating human rights and ethical considerations into training can help nurses understand the broader context of GBV and the importance of respecting victims’ rights. The following supporting statements reflect this:

‘Firstly, it would be basic nursing knowledge taught at different nursing schools and human rights and ethics …. Human rights are the understanding that every individual is subject to Human rights. Every individual has the right to life stipulated in our Constitution, so no individual must be subjected to additions; they are not lawful according to the institutions of our country.’ (Professional Nurse, Participant 4, Male, 32 years)

This sub-theme highlights the importance of grounding GBV management in human rights and ethical principles to uphold victims’ dignity and legal protections. The gap revealed is that while these concepts are introduced in training, there may be insufficient emphasis on their practical application in real-world settings, suggesting a need for more focused, scenario-based ethics education to bridge theory and practice.

## Discussion

Professional nurses face a complex challenge when addressing GBV, balancing the need to respect a victim’s autonomy and confidentiality with their legal and ethical obligations to report specific cases (Jones & Brown 2020:47; Stevens et al. 2024:1). While prioritising victim consent aligns with therapeutic best practices, nurses are also mandated to report specific instances, particularly those involving minors, even without consent (Green & Brown 2018:75). This duality highlights the need for clear guidelines and comprehensive training within HEIs to navigate this challenging landscape with both empathy and legal adherence (White 2019:34). Several support systems are crucial in enabling nurses to respond to GBV effectively. Thuthuzela Care Centres provide comprehensive services, including medical care, legal assistance and counselling, ensuring that victims receive immediate and appropriate support (South African Government 2025; Taylor 2021:23). Nurses’ reliance on these specialised centres underscores the necessity of coordinated care pathways to address the multifaceted needs of GBV victims (Johnson & Stylianou 2022:4). Furthermore, institutional policies and support systems, such as the CSCD, offer essential counselling services and facilitate referrals to other necessary resources (Clark 2021:67). Collaboration with social workers, psychologists and police services exemplifies the interdisciplinary approach required for effective GBV intervention (Adams 2021:45). These institutional frameworks are vital in supporting nurses’ roles and ensuring comprehensive care for victims.

Despite these support systems, challenges remain. While nurses reported using the PACK guidelines to manage GBV cases, inconsistencies in availability and utilisation were noted (Miller 2023:58). This highlights a critical gap in resource provision and training, which can impede nurses’ ability to respond effectively (Wilson 2020). Standardising care and improving victim outcomes require ensuring that all nurses have access to and are adequately trained in the use of these guidelines (CARE-GBV 2022a:4). Effective referral systems to specialised centres and professionals, such as psychologists and social workers, are also paramount (Lewis et al. 2019:310). Interdisciplinary collaboration is essential for providing holistic care to GBV victims, necessitating seamless transitions between different levels of care (Jenney et al. 2023:584).

These findings align with previous research emphasising the importance of interdisciplinary collaboration and robust institutional support in managing GBV (Jones & Brown 2020:45; Stevens et al. 2024:1). Holistic care models that integrate medical, psychological and legal support have consistently proven most effective (Bress et al. 2019:3). While the use of care kits like PACK guidelines is recognised as a best practice, challenges in implementation persist (CARE-GBV 2022b:2). The significance of these findings lies in their potential to inform policy and practice within HEIs. By identifying the strengths and gaps in existing systems, this study provides a basis for developing more effective training programmes and institutional policies supporting professional nurses. The emphasis on interdisciplinary collaboration and resource utilisation underscores the need for integrated care models to improve outcomes for GBV victims. In the broader nursing and public health context, these findings contribute to the ongoing dialogue on enhancing GBV response and prevention strategies.

Professional nurses also develop crucial competencies in recognising and responding to GBV through practical experience (Mann & Lanning 2024:45). This hands-on exposure enables nurses to navigate complex situations and learn practical strategies within their work environment (Cassidy et al. 2021:23). Informal learning, where knowledge is shared among colleagues, fosters a supportive network that collectively enhances understanding and management of GBV cases (Tohidi et al. 2019:93). Approaching GBV victims with empathy and without judgement is also essential for building trust and effectively managing cases (Savarese et al. 2024:8). Effective communication and listening skills are vital for understanding victims’ needs and providing appropriate support (Alhalal 2020:7; Clark 2018:50). Integrating human rights and ethical considerations into training is beneficial for helping nurses understand the broader context of GBV and the importance of respecting victims’ rights (Van Vo et al. 2024:5). Embedding these elements into educational programmes better prepares nurses to handle GBV cases with a comprehensive understanding of the ethical and human rights dimensions (Flaubert, Le Menestrel & Williams 2021:68). The value of practical experience in developing nursing competencies is well supported in the literature (Mann & Lanning 2019:45). Empathy and effective communication are fundamental skills that enhance patient care and support (Haribhai-Thompson et al. 2022:5), while a holistic approach to training that includes ethical dimensions and respect for patient autonomy is crucial (Haddad & Geiger 2023:89).

These findings are significant in the broader context of nursing and healthcare education, highlighting the multifaceted nature of competence development in GBV management. By recognising the value of practical experience, empathy, communication and ethical training, nursing programmes can be better tailored to prepare professionals for the complexities of GBV cases. This enhances nurses’ preparedness and contributes to a more compassionate and effective healthcare response to GBV, ultimately improving outcomes for victims and advancing nursing education.

### Recommendations

The study’s findings and recommendations are intended to guide policy development and encourage educational institutions to implement a more comprehensive curriculum on GBV management, thereby improving the quality of care for affected individuals.

It is crucial that higher education and training institutions embed comprehensive GBV management into nursing undergraduate curricula. Central to this is the creation of a dedicated GBV module that thoroughly addresses the physical, psychological, sexual and reproductive impacts of GBV. This module would fill a critical gap left by recent curriculum reforms, which saw the removal of a specific GBV focus. Covering topics from the prevalence of violence and human rights to legal frameworks and management strategies, the module aims to equip nursing students with the knowledge and skills necessary to identify and support GBV victims effectively.

The processes of analysing curriculum documents, conducting interviews and developing guidelines have been both demanding and insightful.

To keep GBV education relevant and impactful, the recommendations emphasise the importance of regular curriculum reviews and updates. These reviews should focus on overcoming barriers to GBV education, supporting educators in delivering content, adapting the curriculum to evolving community needs, engaging the community and evaluating how curricular changes affect student learning. This ongoing process ensures that nursing programmes remain responsive to the complex realities of GBV and maintain high educational standards.

These guidelines advocate for a comprehensive, evidence-based approach to integrating GBV management into nursing education. By implementing them, institutions can better prepare future nurses to handle the intricacies of GBV cases in healthcare settings. Based on frontline nursing expertise and scholarly research, the recommendations promote a structured, practical framework that strengthens nursing students’ readiness to address GBV with competence and sensitivity.

### Limitation

The study’s limitations include a focus on a specific geographic region, which may limit the transferability of the findings to similar contexts. Additionally, the study relies on participant self-reported data, which may introduce bias. Further research is needed to validate the findings in diverse settings and explore the perspectives of other stakeholders such as policymakers, institutional administrators, healthcare practitioners, public health organisations, community leaders and organisations involved in GBV management.

## Conclusion

The experiences and perceptions of professional nurses reveal the complex challenges they face in addressing GBV within HEIs. They navigate a difficult balance between ethical obligations, legal mandates and prioritising victim autonomy. Despite existing support structures like TCCs and the CSCD, inconsistent availability and utilisation of resources such as the PACK guidelines highlight the need for targeted training and equitable resource allocation to ensure consistent and effective care.

Interdisciplinary collaboration is essential, with seamless referral systems and coordinated efforts among nurses, social workers, psychologists and legal professionals providing holistic support to GBV victims. Developing individual nurse competencies through practical experience, empathy cultivation and embedding ethical considerations into nursing education is equally vital.

This research reaffirms the pivotal role of education as a catalyst for social change and the importance of preparing healthcare professionals to support GBV survivors. Advancing GBV prevention and response within HEIs requires sustained commitment to strengthening institutional frameworks, fostering collaboration and prioritising individual competency development. By addressing identified gaps and building on existing strengths, HEIs can create a more supportive, equitable environment that enhances the well-being of GBV victims and promotes a culture of safety, respect and social justice.

This study contributes to the broader discourse on GBV management by offering actionable insights for curriculum development, policy implementation and care standardisation. By prioritising equitable resource allocation, standardised protocols and seamless referral pathways, HEIs can play a pivotal role in mitigating GBV’s impact, ensuring comprehensive care for victims and fostering a safer academic community. These strategies not only prepare nursing graduates more effectively but also support a wider societal shift towards effective GBV prevention and response.
